# T-Cadherin Expression in Melanoma Cells Stimulates Stromal Cell Recruitment and Invasion by Regulating the Expression of Chemokines, Integrins and Adhesion Molecules

**DOI:** 10.3390/cancers7030840

**Published:** 2015-07-21

**Authors:** Kseniya A. Rubina, Ekaterina I. Surkova, Ekaterina V. Semina, Veronika Y. Sysoeva, Natalia I. Kalinina, Alexei A. Poliakov, Helena M. Treshalina, Vsevolod A. Tkachuk

**Affiliations:** 1 Department of Biochemistry and Molecular Medicine, Faculty of Medicine, M.V. Lomonosov Moscow State University, Lomonosovsky av., 31/5, Moscow 119192, Russia; E-Mails: katerina-u48@mail.ru (K.I.S.); e-semina@yandex.ru (E.V.S.); veroniks@mail.ru (V.Y.S.); n_i_kalinina@mail.ru (N.I.K.); tkachuk@fbm.msu.ru (V.A.T.); 2 Division of Developmental Neurobiology, MRC National Institute for Medical Research, The Ridgeway, Mill Hill, London NW7 1AA, UK; E-Mail: alexei.poliakov@gmail.com; 3 Federal State Budgetary Scietific Institution «N.N. Blokhin Russian Cancer Research Center» (FSBSI “N.N.Blokhin RCRC”), Kashirskoe Shosse 24, Moscow 115478, Russia; E-Mail: treshalina@yandex.ru

**Keywords:** T-cadherin, melanoma, migration, invasion

## Abstract

T-cadherin is a glycosyl-phosphatidylinositol (GPI) anchored member of the cadherin superfamily involved in the guidance of migrating cells. We have previously shown that *in vivo* T-cadherin overexpression leads to increased melanoma primary tumor growth due to the recruitment of mesenchymal stromal cells as well as the enhanced metastasis. Since tumor progression is highly dependent upon cell migration and invasion, the aim of the present study was to elucidate the mechanisms of T-cadherin participation in these processes. Herein we show that T-cadherin expression results in the increased invasive potential due to the upregulated expression of pro-oncogenic integrins, chemokines, adhesion molecules and extracellular matrix components. The detected increase in chemokine expression could be responsible for the stromal cell recruitment. At the same time our previous data demonstrated that T-cadherin expression inhibited neoangiogenesis in the primary tumors. We demonstrate that T-cadherin overexpression leads to the increase in the expression of anti-angiogenic molecules and reduction in pro-angiogenic factors. Thus, T-cadherin plays a dual role in melanoma growth and progression: T-cadherin expression results in anti-angiogenic effects in melanoma, however, this also stimulates transcription of genes responsible for migration and invasion of melanoma cells.

## 1. Introduction

Cadherins are a superfamily of Ca^2+^-dependent adhesion molecules that play an important role in specific cell-cell adhesion, cell recognition and signaling [1]. T-cadherin is a unique member of cadherin superfamily, which lacks transmembrane and cytoplasmic domains and is anchored to the cell membrane via a glycosyl-phosphatidylinositol (GPI) moiety [2]. T-cadherin is located within lipid rafts in the plasma membrane [3] and is redistributed to the leading edge of migrating cells [4]. It is generally accepted that T-cadherin is a signaling receptor involved in transduction of extracellular cues inside the cell rather than an adhesion molecule.

T-cadherin was suggested to be a tumor suppressor factor and its downregulation was associated with tumor growth and metastasis in lung, ovarian, esophageal, bladder cancer, cervical and prostate carcinomas [5]. However, in other cancers such as human invasive hepatocellular carcinoma MAHLAVU, primary osteosarcoma foci, including metastatic lesions and osteosarcoma cell lines, T-cadherin was abundantly expressed [5]. Moreover, recently it was shown that T-cadherin could exert pleiotropic effects on cancer cells *in vivo* and *in vitro*, where both up- or down-regulation of T-cadherin promoted tumor growth but through different mechanisms [6].

Melanoma is known to be the most aggressive form of skin cancer. The functional role of T-cadherin in melanoma progression remains unclear. While T-cadherin is abundantly expressed in keratinocytes, melanocytes and dermal blood vessels in normal human and mouse skin [7,8,9], its expression is diminished in malignant melanoma. We have reported previously that T-cadherin in human melanoma was gradually reduced upon tumor progression: mosaic pattern of T-cadherin expression in primary melanomas was accompanied by partial or complete loss of T-cadherin in melanoma metastasis [10]. While T-cadherin expression was undetectable in 80% of human melanoma cell lines and in migrating melanocyte precursors, 20% of malignant melanomas still expressed T-cadherin [8,10] implying an ambiguous role of T-cadherin in melanoma malignancy. Experiments on re-expression of T-cadherin in human melanoma cells and their transplantation into the *nu/nu* mice exhibited reduced rate of tumor growth *in vivo*, decreased cell capacity for anchorage-independent growth, migration and invasion *in vitro* [8]. At the same time our data indicated that overexpression of T-cadherin in B16F10 mouse melanoma resulted in the increased tumor growth and metastasis in BDF1 mice [10].

Although the mechanism of T-cadherin participation in tumor growth is still unknown, it is most likely that T-cadherin affects tumor progression not only due to its altered expression in tumor cells, but also by non-autonomous influence on tumor neoangiogenesis [7]. We have showed previously that T-cadherin expression in B16F10 melanoma cells leads to inhibition of neovascularization of primary melanoma sites [10].

The present study is a continuation of our previously published work on T-cadherin participation in melanoma progression. Here we demonstrate that anti-angiogenic effects of T-cadherin expression in melanoma cells are due to their increased expression of angiogenic inhibitors and reduced expression of angiogenic activators. As a compensatory reaction melanoma cells produce chemoattractants that activates mesenchymal stromal cells, which are important participants of tumor growth and progression. While in co-culture T-cadherin expressing melanoma cells stimulate stromal cell migration, they exert no effect on stromal cells proliferation. Since there is no T-cadherin shedding into the conditioned medium the effects of melanoma cells on stromal cell activation are mostly paracrine. This is also accompanied by the elevated invasiveness of T-cadherin melanoma cells and their increased production of pro-oncogenic integrins, α3 laminin and protease MMP14.

## 2. Results

### 2.1. Expression of T-cadherin in Mouse Melanoma Cell Clones

**Figure 1:**
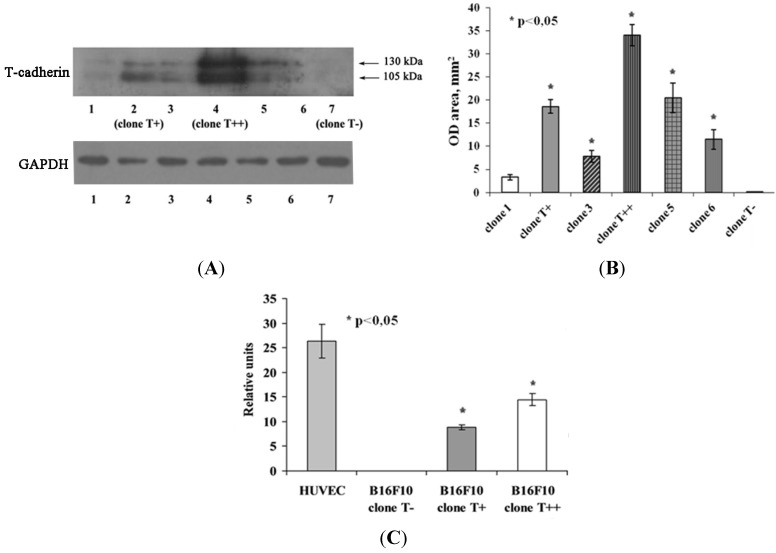
Analysis of T-cadherin expression in B16F10 cell cultures and clones. (**A**) Western blot analysis of T-cadherin expression in B16F10 clones after plasmid transfection. Lanes 2, 4, 7 represent the expression of T-cadherin in B16F10 clones, which were further used for experiments (clone T+, clone T++ and clone T−). Lanes 1, 3, 5 and 6 represent T-cadherin expression in B16F10 clones that were not selected for further experiments. GAPDH was used for loading control. (**B**) Densitometric analysis of T-cadherin western blot bands. For each sample western blots were repeated three times, results are presented as mean ± SEM, * *p* < 0.05 compared to clone T−. (**C**) Real-time PCR analysis of T-cadherin expression in B10F10 clones (clone T+, clone T++ and clone T−). HUVEC cells were used as a positive control. Gene expression was normalized to the expression of 2 housekeeping genes: β-actin and GAPDH. Results are presented as mean ± SEM, * *p* < 0.05 compared to clone T−.

T-cadherin expression after transfection in mouse B16F10 melanoma clones was confirmed by western blot analysis (Figure 1A,B) as well as by quantitative real time PCR (RT PCR, Figure 1C). Three clones of B16F10 melanoma cells with different level of T-cadherin expression were chosen: Control clone with no T-cadherin (clone T−) (lane 7), clone with low T-cadherin expression (clone T+) (lane 2) and clone with high expression (clone T++) (lane 4). Kuphal with co-authors [8] noted that T-cadherin-overexpressing melanoma cell clones lost their expression over a period of time in culture. Therefore, we verified T-cadherin expression in melanoma clones before each experiment.

### 2.2. Effect of B16F10 Clones on Mouse Adipose Derived Stromal Cells Migration in Co-Culture Experiments

**Figure 2:**
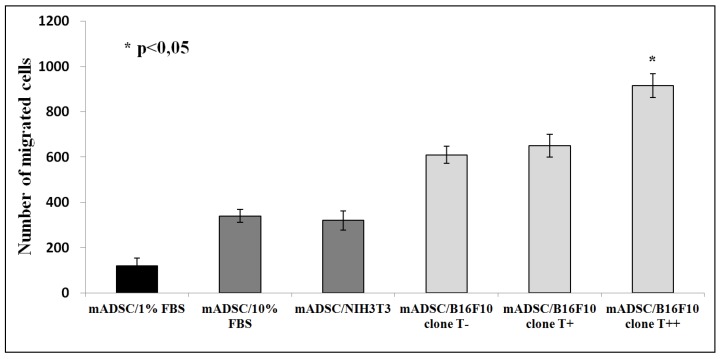
Effect of conditioned medium from B16F10 cell clones (clone T−, clone T+ and clone T++) on migration of mADSCs. Migrated mADSCs were calculated after fixation and hematoxylin staining. The results shown are mean ± SEM of 3 independent experiments performed in duplicates (* *p* < 0.05).

To understand cellular and molecular mechanisms behind the *in vivo* effects of T-cadherin-mediated recruitment of mesenchymal stromal cells to the growing tumor site, we utilized the Transwell^TM^ migration assay. Mouse adipose derived stromal cells (mADSCs) were seeded in the upper chamber and allowed to migrate in the transwell system through collagen-coated membrane to the lower chamber with cultured melanoma cells. The conditioned medium from the clones served as a chemoattractant for mADSCs. We found that migration of mADSCs towards clone T++ was at least 1.5-folds more than towards T+ or T− melanoma clones (*p* < 0.05) (Figure 2).

**Figure 3:**
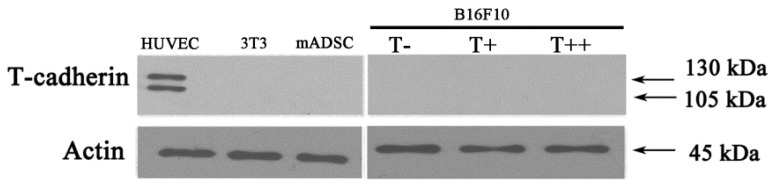
Western blot analysis of T-cadherin shedding into conditioned media from B16F10 melanoma clones (T−, T+ and T++), 3T3 fibroblasts and mADSC. Whole cell lysates from HUVEC cells were used as a positive control. Western blots were repeated three times for three independent experiments. No T-cadherin was detected in the conditioned media.

No shedding of T-cadherin into conditioned medium from melanoma clones (B16F10 T−, B16F10 T+ and B16F10 T++) could be detected as revealed by western blot analysis (Figure 3).

### 2.3. Effect of B16F10 Clones on mADSC Proliferation in Co-Culture Experiments

**Figure 4:**
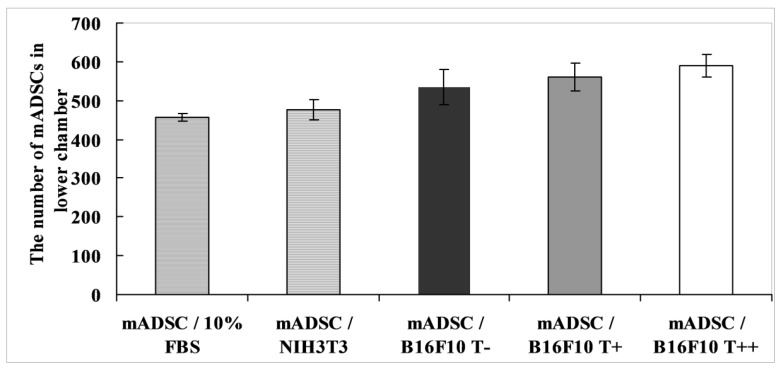
Effect of conditioned medium from B16F10 cells on proliferation of mADSCs. The number of cells was calculated after fixation and hematoxylin staining. NIH3T3 stands for 3T3 fibroblasts. The results shown are mean ± SEM of 3 independent experiments performed in duplicates.

Co-culture experiments using transwell system revealed that conditioned media from T-cadherin-expressing melanoma clones (B16F10 T−, B16F10 T+ and B16F10 T++) had no effect on mADSCs proliferation compared to positive control FBS-containing medium or conditioned medium from NIH/3T3 fibroblasts (Figure 4).

In the present paper we support our previously published *in vivo* results: conditioned media from T-cadherin expressing melanoma cells stimulated mADSCs migration, while exerting no effect on mADSCs proliferation *in vitro*.

### 2.4. Effect of T-cadherin Expression on Invasiveness of B16F10 Melanoma Clones in Vitro

**Figure 5:**
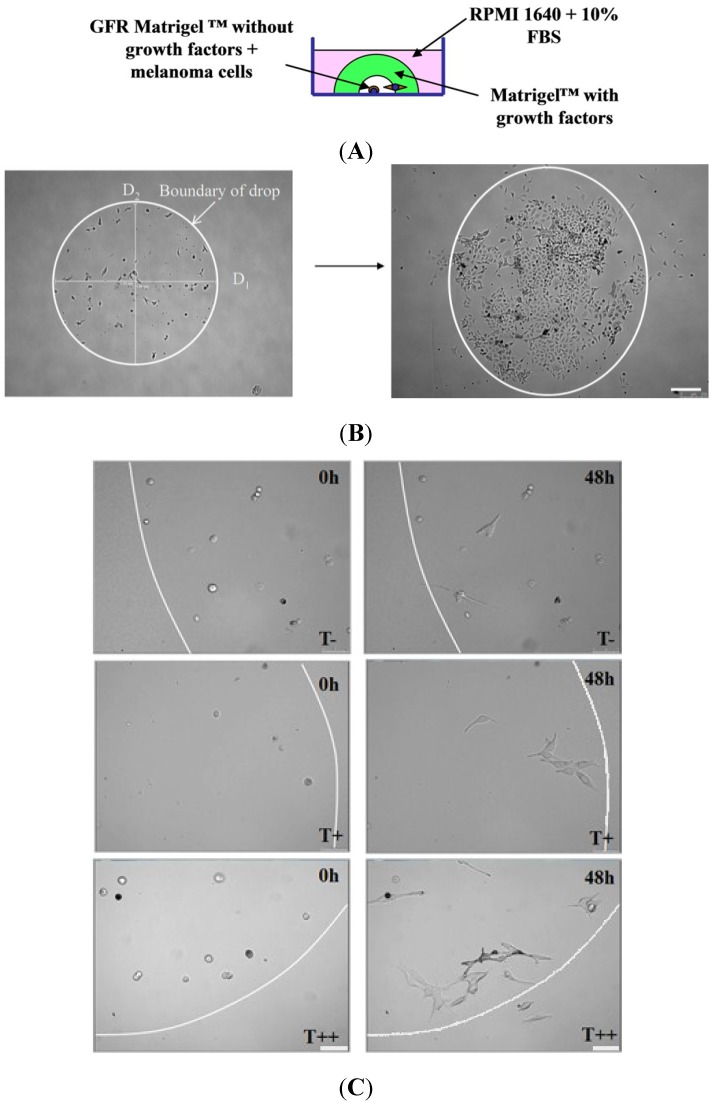
Experimental design (**A**) and phase contrast images of Matrigel drops (**B**), (**C**) with melanoma cells. The demarcation line (**B**), (**C**) shows the border between two Matrigels (Matrigel GFR Matrigel^TM^—Growth factor reduced Matrigel and Matrigel with growth factors). (**B**) A representative Matrigel drop, containing melanoma cells at the beginning of the experiment and 48 h after (**B**). Bars 250 μm. (**C**) Images of Matrigel drops (T−, T+ and T++ melanoma clones) at the onset of the experiment (0 h) and after 48 h (48 h). Bars 50 μm.

Invasive potential of melanoma clones was assessed by counting the number of cells that invaded from growth factors reduced (GFR) Matrigel into complete Matrigel in a three-dimension invasion assay specifically designed and described in Figure 5. The videos demonstrating melanoma cell invasion in Matrigel drop assay (T−, T+, and T++ clones) are presented in the Supplementary Materials.

**Figure 6:**
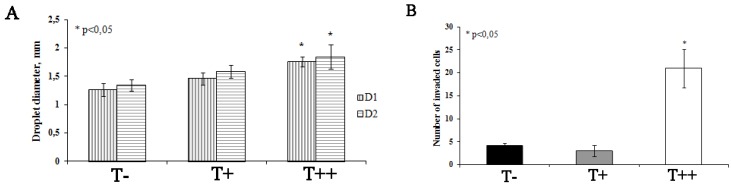
Effect of T-cadherin expression on invasive capacity of B16F10 melanoma clones (T−, T+ and T++) *in vitro*. (**A**) Quantitative evaluation of the change in two diameters (D1 and D2) of the inner Matrigel drops (**B**). Quantitative evaluation of the number of invaded melanoma cells from GFR Matrigel into complete Matrigel. The results shown are mean ± SEM, * *p* < 0.05 compared to clone T−.

Melanoma invasion was accompanied by a proteolytic cleavage of matrix proteins by migrating cells. As a result, the initially well-defined boundaries between two Matrigels became blurred (but still detectable) several hours after the onset of the experiment. This was also convoyed by the change in the inner diameter of the Matrigel drop (Figure 5). We evaluated two diameters of the Matrigel inner drop and found that after 48 h after the beginning of the experiment, both diameters were significantly larger in Matrigels with clone T++ clone, compared to T+ and T− clones. These data suggested that clone T++ had a significantly higher proteolytic activity compared to T+ or T− clones (Figure 6A). Moreover, the number of T++ cells that invaded into the complete Matrigel was 5–7-fold higher compared to T+ or T− cells (Figure 6B). Taken together, these results indicated that overexpression of T-cadherin in melanoma cells resulted in the increased invasive potential. The corresponding lifetime observations of invading melanoma cells are presented in the supplementary materials.

### 2.5. Effect of T-cadherin on Gene Expression

To reveal the possible molecular mechanisms underlying the effects of T-cadherin on tumor growth and metastasis that have been shown before [10] we carried out PCR Array analysis. On angiogenic array, it was found that in T++ and T+ clones the expression of three genes was highly upregulated (greater than 4-fold) (Table 1). The most upregulated genes were two antiangiogenic molecules: CXCL10 and heparanase. T-cadherin expression resulted in 2-fold upregulation of angiopoietin 2 and stabilin 1 which are also anti-angiogenic molecules. The expression of two genes, chromogranin A and procollagen XVIIIα1, precursors of angiogenic inhibitors, was detected only in T-cadherin-expressing B16F10 cells (T+ and T++). Interestingly, the expression of 2 proangiogenic molecules Tie1 and TGFα was essentially inhibited in T++ and T+ B16F10 clones, while the remaining 75 genes on the array were not significantly affected.

**Figure 7:**
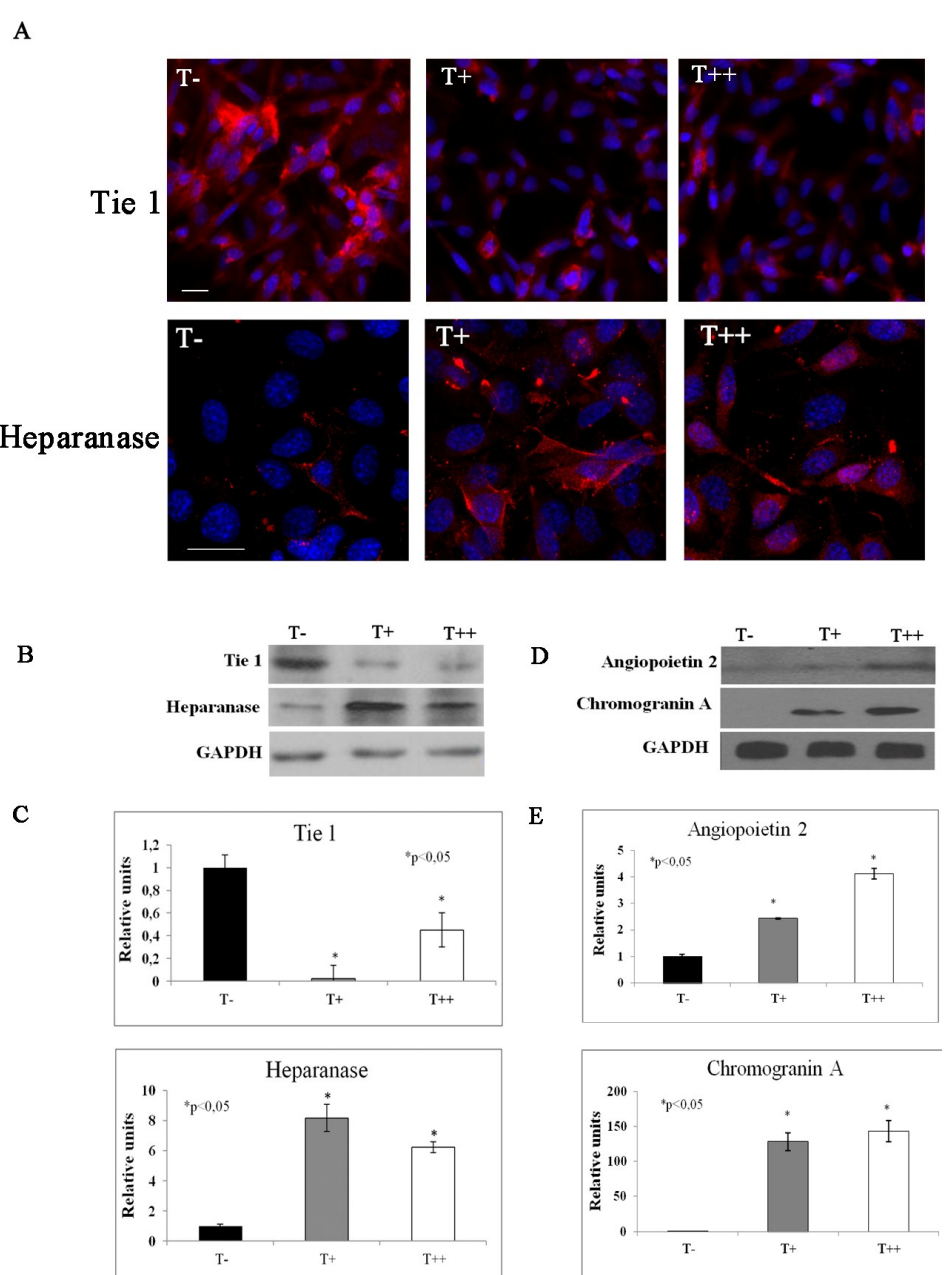
Immunofluorescent staining with high resolution confocal imaging (**A**) and western blots (**B**) and (**D**) with densitometric analysis (**C**) and (**E**) of B16F10 melanoma clones. Images of control cells (T−) and cells expressing T-cadherin (T+ and T++) were obtained after immunofluorescent staining with antibodies against Tie 1 or Heparanase (red fluorescence). Nuclei appear blue after DAPI staining. Bars 10 μm. Protein expression of tie 1, angiopoetin 2, heparanase and chromogranin in T−, T+ and T++ cell lysates were detected by western blot. For each clone western blots were repeated three times, * *p* < 0.05 compared to clone T−. Results are the means ± SEM.

To confirm the results of angiogenic RT PCR Array we performed immunofluorescent staining with confocal microscopy image analysis as well as western blot with densitometry. Immunofluorescent staining confirmed that T-cadherin overexpression in melanoma clone culture resulted in the reduced expression of Tie 1 and increased expression of heparanase (Figure 7A). As demonstrated by western blot these also corresponded to the upregulation of heparanase, angiopoietin 2 and chromogranin A expression and downregulation of Tie 1 in T++ cells (Figure 7B,C).

**Table 1:** Angiogenic RT PCR Array results. Data represents fold-change in gene expression in T+ and T++ melanoma cells relative to control cells. Per experiments, a set of five housekeeping genes (GUSB, HPRT1, HSP90AB1, GAPDH, ACTB) was included.

Fold Regulation Compared to Control Clone		Gene
B16F10 Clone T+	B16F10 Clone T++	
−10	−11	Tyrosine kinase receptor 1 (Tie 1)
2	2	Angiopoietin 2
2	3	Stabilin 1
4	5.5	Forkhead box F1a (Foxf1a)
5	7.8	Chemokine (C-X-C motif) ligand 10 (CXCL10)
8.7	6.3	Heparanase

The most highly upregulated gene on the chemokine array (greater than 10-fold) was CXCR7 (Table 2). The expression of chemokine CXCL10 was also upregulated (greater than 4-fold). Upon T-cadherin expression five additional genes were found to be at least 2-fold upregulated including CCL5, CMTM3, Nfkb1 (p105), CXCL11 and CCRL1. The expression of three genes (CCL7, Gdf 5 and interleukin 8rα) was detected only in T-cadherin-expressing clones, while interleukin 16, interleukin 4 and interleukin 8rβ were found only in the control clone. Noteworthy, in T++ and T+ melanoma clones the expression of chemokine CCL9 was also significantly inhibited, while the remaining 74 genes on the array were not substantially affected.

**Table 2:** Chemokine RT PCR Array results. Data represents fold-change in gene expression in T+ and T++ melanoma cells relative to control cell. Per experiments, a set of five housekeeping genes (GUSB, HPRT1, HSP90AB1, GAPDH, ACTB) was included.

Fold Regulation Compared to Control Clone		Gene
B16F10 Clone T+	B16F10 Clone T++	
−1,4	−3.1	Chemokine (C-C motif) ligand 9 (CCL9)
1.5	2	Chemokine C-C motif receptor-like 1 (CCRL1)
1.8	2.5	Chemokine (C-X-C motif) ligand 11 (CXCL11)
2	3.2	Nuclear factor of kappa light chain gene enhancer in B cells, p105 (Nfkb1)
2.4	2.2	CKLF-like MARVEL transmembrane domain containing 3 (CMTM3)
3.3	2.4	Chemokine (C-C motif) ligand 5 (CCL5)
2.9	15.3	Chemokine (C-X-C motif) ligand 10 (CXCL10)
5.7	17.6	Chemokine C-X-C motif receptor 7 (CXCR7)

**Figure 8:**
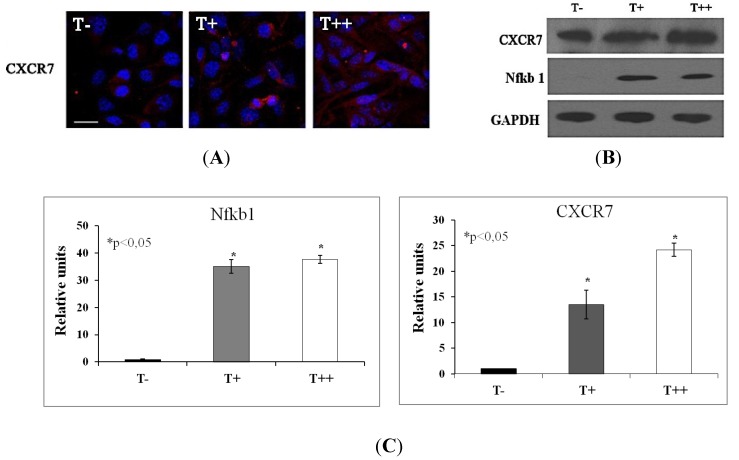
Immunofluorescent staining with high resolution confocal imaging (**A**) and western blot (**B**) with densitometric analysis (**C**) of B16F10 melanoma clones. Images of control cells (T−) and cells expressing T-cadherin (T+ and T++) were obtained after immunofluorescent staining with antibodies against CXCR7 (red fluorescence). Nuclei appear blue after DAPI staining. Bars 10 μm. Protein expression of CXCR7 and Nfkb1 (p105) in T−, T+ and T++ cell lysates were analyzed using western blot. For each sample Western blots were repeated three times, * *p* < 0.05 compared to clone T−. Results are the means ± SEM.

To confirm the results of chemokine RT PCR Array we carried immunofluorescent staining with confocal microscopy and western blot with densitometry. It was shown that T-cadherin expression resulted in the increased production of CXCR7 (Figure 8A) and Nfkb1 (p105) proteins (Figure 8B,C), which correlates with RT PCR Array data.

Using the extracellular matrix and adhesion molecule RT PCR Array analysis, we found that the expression of cadherin 1 (Table 3) was upregulated more than 10-fold in T++ melanoma cells. 8 additional genes were activated at least 2-fold in T++ and T+ B16F10 clones, including procollagen Iα1, MMP14, fibronectin 1, TGFbi and four integrins: α5 (fibronectin receptor alpha), β3, α5 αE, αV. It should be noted that expression of 2 genes: Laminin α3 and PECAM 1 was detected only in T-cadherin-positive melanoma cells. While the expression of adamts 5 was inhibited in T-cadherin-expressing clones; the remaining 70 genes on the array were not significantly affected.

**Table 3:** Adhesion molecule RT PCR Array results. Data represents fold-change in gene expression in T+ and T++ melanoma cells relative to control cell. Per experiments, a set of five housekeeping genes (GUSB, HPRT1, HSP90AB1, GAPDH, ACTB) was included.

Fold Regulation Compared to Control Clone		Gene
B16F10 Clone T+	B16F10 Clone T++	
−2.3	−2.1	A distintegrin-like and metallopeptidase (reprolysin type) with thrombospondin type 1 motif, 5 (Adamts 5)
1	3.3	Integrin alpha E, epithelial-associated
1.1	2.3	Integrin beta 3
1.2	3.9	Fibronectin 1
1.3	2.6	Integrin alpha 5 (fibronectin receptor alpha)
1.3	3.2	Matrix metallopeptidase 14 (MMP14)
1.8	2.5	Integrin alpha V
2.3	2	Transforming growth factor, beta induced (TGFbi)
4	15	Cadherin 1

**Figure 9:**
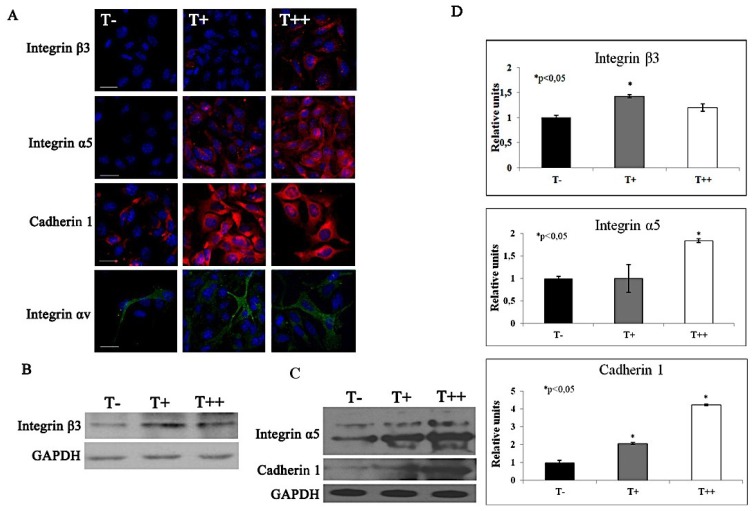
Immunofluorescent staining with high resolution confocal imaging (**A**) and western blots (**B**), (**C**) with densitometric analysis (**D**) of protein expression in B16F10 melanoma clones. Images of control cells (T−) and cells expressing T-cadherin (T+ and T++) were obtained after immunofluorescent staining with antibodies against integrin β3, integrin α5, cadherin 1 (red fluorescence) and integrin αv (green fluorescence). Nuclei appear blue after DAPI staining. Bars 10 μm. Protein expression of integrin β3, integrin α5 and cadherin 1 in T−, T+ and T++ cell lysates were detected by western blot. For each sample western blots were repeated three times, * *p* < 0.05 compared to clone T-. Results are the means ± SEM.

We confirmed the results of PCR Array analysis utilizing immunofluorescent staining with confocal microscopy and western blot with densitometry. Upon T-cadherin expression we verified the upregulation of integrin β3, integrin α5, cadherin 1 (Figure 9A–C) and integrin αV (Figure 9A).

## 3. Discussion

Anti-angiogenic properties of T-cadherin have been demonstrated *in vivo* and *in vitro* in our lab before. Using a Matrigel implant model in mice, we have shown that subcutaneous injection of Matrigel, containing T-cadherin-expressing L929 cells inhibited neovascularization of the plugs [11]. This was also confirmed using chorioallantoic membrane of chick embryo where melanoma masses formed by T-cadherin expressing cells showed decreased vascularization [12]. Quantitative analysis of blood vessels formed by B16F10 melanoma cells with different level of T-cadherin expression confirmed that T-cadherin suppresses blood vessel formation in the primary tumors [10]. Other research groups have demonstrated that T-cadherin could be upregulated in blood vessels penetrating lung metastases, mouse mammary tumors and human hepatocellular carcinoma with reduced expression of T-cadherin in tumor cells [5,6,13]. All these data support the notion that T-cadherin is a negative regulator of blood vessel growth and that the mechanism of contact inhibition might be responsible for these T-cadherin effects [11].

While the blood vessel growth is tightly controlled under physiological conditions, tumor progression is frequently associated with intensive neoangiogenesis induced by a switch in the balance of pro- and anti-angiogenic molecules [14]. In the present study the PCR Array data indicate that T-cadherin expression in B16F10 melanoma cells results in the activation of genes of such angiogenic inhibitors as procollagen type XVIIIα1 (a precursor of the angiogenesis inhibitor endostatin) [15] and chromogranin A (a precursor of angiogenesis inhibitor vasostatin-1) [16] which have not been detected in control cells. T-cadherin expression also leads to upregulation of mRNA encoding anti-angiogenic molecules such as stabilin 1, heparanase, CXCL10 [17] and angiopoietin 2 [18]. T-cadherin expressing cells also demonstrate a decreased expression of such pro-angiogenic molecules as TGFα [19] and Tie 1 [20]. Interestingly, PECAM 1 (CD31) expression was detected only in T-cadherin-positive clones suggesting the possible mechanism of “vascular mimicry” [10] and compensatory reaction of melanoma cells to reduced expression of angiogenic factors. The PCR data on heparanase, angiopoietin 2, chromogranin A and Tie 1 has been confirmed at the protein expression level using immunofluorescent staining with confocal microscopy and western blot analysis. Thus, the data obtained in the present study indicate that the balance between angiogenic inducers and inhibitors is switches towards inhibitors upon T-cadherin expression, which might be responsible for the reduced neovascularization in mice *in vivo* reported before [10].

In some tumors, the decline of oxygen tension has been shown to act as a selective pressure that leads to cell proliferation with increased metastatic potential. Since the invasive and metastatic activity reflects the ability of melanoma cells to degrade the matrix and migrate along the gradient of chemoattractants we evaluated the invasion potential of melanoma cells and their expression of proteases, pro-oncogenic integrins and some chemokine receptors. Here we’ve shown that T-cadherin expression results in the enhanced migratory and invasive potential of melanoma cells.

Apparently, the invasive and metastasizing cancer cells are characterized by changes in integrin expression pattern [21]. Since overexpression of integrins such as ανβ3, αβ5, αβ1, αβ1, αIIβ3 and αβ1 or their single subunits has been correlated with the transition from primary melanoma to metastatic [22], we compared the expression level of various integrins in control and T-cadherin expressing melanoma cells. The PCR array results demonstrate the upregulation of mRNA encoding integrins α5, αV, αE, and β3 upon T-cadherin overexpression. This upregulation of integrin β3, integrin α5, integrin αV and cadherin 1 at mRNA level has been confirmed by immunofluorescent staining with confocal microscopy and western blot. These data correlate with the increase in invasive potential of these cells shown in 3D invasion test (Figure 5).

Melanoma cells are known to produce multiple isoforms of laminin and mediate their attachment and invasion via integrin receptors using laminin as a substrate [23]. Moreover, there is strong evidence that expression of fibronectin is tightly correlated with the acquisition of invasive and metastatic behavior of melanoma [24]. Noteworthy, the elevated level of mRNA expression of fibronectin 1 was found in T+ and T++ melanoma cells, while laminin α3 was detected only in T-cadherin expressing cells, suggesting their important role in the increased invasive potential of these cells.

It is known that overexpression of some integrins can stimulate matrix metalloproteinases (MMPs) activity in melanoma cells, which contributes to their invasive capacity [25,26]. Several MMPs, including MMP-1, -2, -3, -7, -9, -13, -14, -15, -16 as well as uPA have been demonstrated to play a crucial role in human melanoma progression, invasion and metastasis [27]. The PCR Array analysis in the present study indicates that the only protease, which expression has increased upon T-cadherin overexpression was MMP14.

Accumulating evidence indicates that tissue-specific gradient of chemokines and the expression profile of chemokine receptors on cancer cells determine the site and pattern of metastases of many tumors [24]. In a screen of several melanoma cell lines it was detected that the expression of chemokine receptors CCR7, CCR10, CXCR1, CXCR2 and CXCR4 can dramatically increase the rate of metastases and define their preferential site [28,29,30,31]. The expression of chemokines such as CCL5, CXCL10 and others were associated with malignant melanoma progression [31]. Our PCR array analysis indicates that expression of three genes such as CCL7, Gdf 5 and interleukin 8rα was detected only in T+ and T++ melanoma clones. The expression of chemokines and chemokine receptors such as CXCL10, CXCL11, CXCR7, CXCR1 and CCL5 was upregulated in T-cadherin-overexpressing cells, which correlates with their enhanced invasive and metastatic activity *in vivo* [10] and *in vitro*.

Previously, we demonstrated that T-cadherin expression in B16F10 melanoma cells decreased the growth of tumors formed on chorioallantoic membrane in chick embryo [12]. However, our recent study indicated that mice bearing tumors formed by T-cadherin expressing B16F10 cells showed lower survival due to the increased primary tumor growth and metastasis. While proliferation of B16F10 melanoma cells *in vitro* was not affected by T-cadherin expression, the increase in the primary tumor volume in mice was considerable. Imunofluorescent staining of these tumor sections indicated that T-cadherin expression in melanoma cells potentiated the recruitment of CD90-positive mesenchymal cells into the primary tumor sites [10]. We suggest that the difference in these two experimental models may partly account for these results. As such the chorioallantoic membrane of chick embryo is the model where there could be no contribution of MSCs into the murine melanoma tumor growth due to the species specificity. In contrast, subcutaneous transplantation of melanoma cells of murine origin into mice could lead to specific reaction of the cellular environment and stimulate stromal cell migration into the primary tumor site.

In this paper we confirm our previously published *in vivo* results: T-cadherin exerts stimulating effects on the surrounding stromal cells [10]. No T-cadherin was detected in the conditioned medium from T-cadherin expressing cells as tested by western blot analysis. The conditioned medium from T-cadherin expressing melanoma cells stimulated mADSCs migration in transwell system, while had no effect on mADSCs proliferation *in vitro*. Thus we speculate that the stimulating effects of melanoma cells were mostly paracrine due to the production of chemokines.

The obtained data are fundamental for understanding the mechanisms of invasive melanoma progression underlying the dissemination process and low sensitivity to the drug therapy. Detected by microarray and confirmed by immunochemistry and western blot, properties of T-cadherin do not allow us to consider this protein a tumor suppressor. Along with the inhibition of vessel growth, T-cadherin stimulates the transcription of genes responsible for survival, migration and invasion of melanoma cells.

## 4. Materials and Methods

### 4.1. Cell Culture and Generation of Stable Cell Lines

Murine malignant melanoma B16F10 cell line was obtained from ATCC (ATCC^®^ No CRL-6475™). Cells were cultured in RPMI 1640 medium (HyClone, Logan, UT, USA), with 10% FBS (HyClone), 100 U/mL penicillin (HyClone), 100 μg/mL streptomycin (HyClone), 0.25 μg/mL amphotericin B (HyClone), 2 mM L-glutamine (HyClone).

As previously described [11], full-length human T-cadherin cDNA was cloned in pcDNA™ 3.1 vector (Invitrogen, Waltham, MA, USA) to generate pcDNA-Tcad. B16F10 cells were transfected using pcDNA-Tcad with Lipofectamine™ 2000 reagent (Invitrogen) to generate T-cadherin-expressing cells. For control, luciferase cDNA fragment in antisense orientation was cloned into the pcDNA 3.1 vector. 96 h after transfection cells were cloned in RPMI1640/10% FBS with 2 mg/mL G418 (Invitrogen) in 96-well plates using limiting dilution method.

### 4.2. Preparation of Tumor Conditioned Media

B16F10 cells (with concentration 1 × 10^5^/well) of each clone were seeded in 100 mm Petri dish and incubated for 48 h in serum-free RPMI1640 media. The conditioned medium from clones of B16F10 cells was collected and concentrated with a spin-column Centricon 30 kDa (Millipore, Billerica, MA, USA) and stored with protease inhibitor cocktail (1:100, Sigma-Aldrich, St. Louis, MO, USA) at −20 °C. The conditioned medium from equivalent condition of NIH3T3 fibroblasts were used for control.

### 4.3. Western Blot

Expression of T-cadherin, chemokines, adhesion and angiogenic molecules in cell lysates as well as T-cadherin shedding into conditioned media of B16F10 clones was analyzed. Total lysates of B16F10 clones were obtained in 200 μL of lysis buffer (50 mMTris/HCl, pH 8 (Sigma-Aldrich, St. Louis, MO, USA), 5 mM EDTA (AppliChem, Darmstadt, Germany), 1% Triton X-100 (PRS Panreac, Barcelona, Spain), 20 mM HEPES, 150 mM NaCl (PRS Panreac) and protease inhibitor cocktail (Sigma-Aldrich). Protein concentration was determined by Bradford assay. Samples were diluted to equivalent protein concentrations in Laemmli buffer, containing β-mercaptoethanol (Helicon, Moscow, Russia). 30 μg of supernatant protein were loaded per lane and separated using 7.5% SDS/polyacrylamide gel. Samples were electroblotted onto Immobilon PVDF membranes (Millipore, Billerica, MA, USA) and incubated with primary antibodies rabbit anti mouse T-cadherin antibody 1:1000 (Biodesign International, Saco, ME, USA), rabbit anti-mouse actin antibody (Sigma-Aldrich), rabbit anti mouse heparanase 1 antibody 1:1000 (Abcam, Cambridge, MA, USA), rabbit anti mouse angiopoietin 2 antibody 1:1000 (Abcam), rabbit anti mouse chromogranin A antibody 1:1000 (Abcam), rabbit anti mouse Tie 1 antibody 1:2000 (Santa Cruz Biotechnology, Santa Cruz, CA, USA), rabbit anti mouse Nfkb 1 1:500 (Abcam), rabbit anti mouse CXCR7 antibody 1:100 (Lifespan Biosciences) rabbit anti mouse integrin alpha 5 1:500 (Abcam), rabbit anti mouse integrin beta 3 1:1000 (Abcam), rabbit anti mouse cadherin 1 antibody 1:3000 (Abcam), 1:10000 rabbit anti-GAPDH (Santa Cruz), rabbit anti-actin 1:1000 (Santa Cruz) and with secondary antibodies conjugated with horseradish peroxidase (1:10,000, Immune Jackson Research, West Grove, PA, USA). Signals were detected using the ECL system (Amersham Bioscience, Piscataway, NJ, USA). Densitometric analysis of blots was performed using GS-800 Calibrated Densitometer (BioRad, Hercules, CA, USA) and Quantity One 4.6 Software (BioRad). Expression of T-cadherin was normalized to the total protein level.

### 4.4. Quantitative RT PCR

The RNeasy Mini Kit (Qiagen, Hilden, Germany) was used for extraction of total RNA. cDNA were prepared using the RevertAid H Minus First Strand cDNA Synthesis Kit (Fermentas, Waltham, MA, USA). The primers were obtained from Sintol (Moscow, Russia) (Table 4). Real-time qPCR analysis was performed with SYBR Green I on Rotor—Gene™ 3000 (Corbett Research, Cambridgeshire, UK). Gene expression was normalized to the expression of 2 housekeeping genes: β-actin and GAPDH. Primer specificity was confirmed by melt curve analysis. The qRT-PCR was repeated five times.

**Table 4:** Sequences of primers used in qRT-PCR.

Gene	Annealing Temperature	Forward 5′-3′	Reverse 5′-3′
GAPDH	60	GACCCCTTCATTGACCTCAACTAC	TGGTGGTGCAGGATGCATTGCTGA
β-actin	61	AGTGTGACGTTGACATCCGTA	GCCAGAGCAGTAATCTCCTTCT
T-cadherin	60	TTCAGCAGAAAGTGTTCCATAT	GTGCATGGACGAACAGAGT

### 4.5. RT PCR Array Assay

Angiogenesis-specific gene expression, expression of extracellular matrix molecules, adhesion molecules and chemokine gene profiles were analyzed using the PCR Array assay (SABiosciences, Valencia, CA, USA).

Total RNA was isolated from melanoma clones using RNeasy Mini kit. 1 μg of total RNA was treated with DNase, cDNA was prepared using RT^2^ First Strand kit (SABiosciences). For each experiment, cDNA sample was mixed with RT^2^ qPCR Master mix and distributed across the PCR array 96-well plates. After cycling using real-time PCR (IQ5 PCR platform, BioRad), obtained amplification data (fold-changes in C_t_ values) was analyzed with SABiosciences software. Five endogenous control genes: β-glucuronidase (*GUSB*), hypoxanthine phosphoribosyltransferase (*HPRT1*), heat shock protein 90 kDa alpha (cytosolic), class B member 1 (*HSP90AB1*), glyceraldehyde-3-phosphate dehydrogenase (*GAPDH*), and β-actin (*ACTB*) present on the PCR array were used for normalization. Each replicate cycle threshold (*C*_T_) was normalized to the average CT of five endogenous controls on a per plate basis according to the manufacturer’s instructions. The relative expression of each gene, compared to expression in control clone, was calculated on the website using ∆∆Ct method (ΔΔ*C*_T_ = Δ*C*_T_ (T-cadherin expression) − ΔC_T_ (control)). *C*_T_ was defined as 35 for the Δ*C*_T_ calculation when the signal was below detectable limits. Difference in gene expression between T-cadherin-transfected cells and vector control were illustrated as a fold increase/decrease. Gene was considered differentially regulated if the difference was >2-fold compared to the control clone. The RT PCR Array Assay was repeated three times.

### 4.6. Immunofluorescence and Confocal Imaging

B16F10 melanoma cells were seeded on glass coverslips in concentration 2 × 10^4^ cells/cm^2^ and grown 24 h prior to staining. For intracellular antigen staining cells were washed with warm HBSS, fixed with 4% paraformaldehyde, permeabilized for 5 min with 0.2% Triton X-100 (if unless otherwise indicated), and blocked with 10% normal donkey or goat serum with 1% BSA. After sequential incubation with primary and secondary antibodies according to manufacturer’s instructions, coverslips were stained with DAPI (Invitrogen) for nuclei staining and mounted in ProLong^®^ Antifade reagent. First antibodies used: Rabbit anti mouse Heparanase 1:100 (Abcam), rabbit anti Tie 1 1:100 (Abcam), rabbit anti mouse CXCR7 1:150 (Lifespan), rabbit anti mouse cadherin-1 1:100 (Santa Cruz), rabbit anti mouse Integrin α5 1:70 (Abcam), rat anti mouse Integrin αV 1:50 (Abcam), rabbit anti mouse Integrin β3 (Santa Cruz). Images were acquired by confocal laser scanning microscopy system (TCS SP5, Leica) equipped with a Plan-Apo ×60, 1.40 NA oil objective and 488- and 543-Argon lasers lines. Imaging was performed at room temperature using Leica Type F immersion oil. DAPI, AlexaFluor^®^488 and AlexaFluor^®^594 fluorescence was sequentially excited using lasers with wavelengths of 405 (DAPI), 488 (AlexaFluor^®^488) and 594 (AlexaFluor^®^594). All images were captured with the same confocal gain and offset settings. Images (2048 × 2048 pixels) were saved as TIFF files in Leica LAS software and then complied in Adobe Photoshop version CS5 (Softonic Internacional S.A., San Francisco, CA, USA). Results of at least three independent experiments are presented.

### 4.7. Three-Dimension Invasion Assay in Vitro

To evaluate the invasive capacity of melanoma cells, we developed the two-phase three-dimension Matrigel assay (Figure 6). The assay was designed to evaluate the ability of cells to migrate and degrade the matrix moving from the Growth Factor Reduced (GFR) Matrigel into complete Matrigel along the gradient of chemoattractants. Melanoma clones were serum-deprived for 24 h. 3 × 10^5^ cells/mL in RPMI 1640 medium were mixed with GFR Matrigel (BD Biosciences, San Jose, CA, USA) (1:1) and 0.2 μL drops of this mixture were applied on the Petri dishes. Polymerization was induced by incubation at 37 °C for 2 min. After that the preformed drops were covered with 5 μL of complete Matrigel containing the growth factors (BD Biosciences). Polymerization was induced by incubation at 37 °C for 2 min. Then RPMI 1640/10% FBS medium was added into each dish and cells were incubated at 37 °C for 48 h. The drops were photographed after 24 and 48 h using Leica microscope AF6000 (Leica Microsystems, Wetzlar, Germany). The diameters of the Matrigel drops were measured using LasAF software (Leica Microsystems). The number of cells that invaded from GFR Matrigel into complete Matrigel was calculated. For each clone the assay was performed in 25 drops and was repeated three times.

### 4.8. Transwell^TM^ Co-Culture Experiments

To evaluate the effect of T-cadherin expression in melanoma cells on their profile of secreted factors, their ability to stimulate the migration and proliferation of mesenchymal stromal cells we used transwell system. The transwell system allows the exchange of the medium between the lower and the upper chambers however there is no direct cell-cell interaction. Mouse adipose-derived stromal cells (mADSCs) were isolated as previously described [10].

In order to assess the ability of melanoma cells to stimulate migration of mADSCs, the mesenchymal cells were seeded (10^4^/well) in RPMI 1640/0.1% FBS in the upper chamber of transwells onto the 8-μm pore polycarbonate membrane (Transwell, Corning Inc., New York, NY, USA), coated with collagen I (0.1 μg/mL, IMTEK, Moscow, Russia). Melanoma cells were serum-deprived for 16 h and seeded in the lower chamber (5 × 10^4^ cell/well). Conditioned medium from melanoma clones in the lower chamber served as a chemoattractant for migrating mADSCs. Cells were incubated at 37 °C for 48 h. Nonmigrated mADSCs were removed gently by wiping the upper surface of the membrane with a cotton swab. Membranes were fixed in 4% formaldehyde (PRS Panreac) and stained with Mayer’s hematoxylin (Dako, Carpinteria, CA, USA). Migration was assessed by counting the number of mADSCs present on the lower surface of the membrane using light microscope. Control wells contained NIH/3T3 cells or RPMI1640/10% FBS medium in the lower chamber.

To assess the effect of melanoma cells on proliferation of mADSCs, the latter cells were seeded (10^4^/well) in RPMI 1640/0.1% FBS in the lower chamber of transwells with 0.4-μm pore polycarbonate membrane (Transwell). Melanoma cells were serum-deprived for 16 h and seeded in the upper chamber (5 × 10^3^ cell/well). Cells were incubated at 37 °C for 48 h. mADSCs in the lower chamber were fixed in 4% formaldehyde (PRS Panreac, Barcelona, Spain) and stained with Mayer’s hematoxylin (Dako). The number of mADSCs in the lower chamber was assessed using light microscope. Control wells contained NIH/3T3 cells or RPMI1640/10% FBS medium in the upper chamber.

### 4.9. Statistics

Statistical analysis was performed using Statistica 6 software (StatSoft, Tulsa, OK, USA). Results are presented as mean ± SEM. All parameters were evaluated by the non-parametric Mann-Whitney test. Differences with *p* < 0.05 were considered as statistically significant.

## 5. Conclusions

We provide experimental evidence for a potentially dual role of T-cadherin in tumor progression. Detected by microarray and confirmed by immunochemistry and western blot, T-cadherin overexpression results in the increased invasive potential due to the upregulated expression of pro-oncogenic integrins, chemokines, α3 laminin and MMP14. However, T-cadherin overexpression leads to the increase in anti-angiogenic molecules and reduction in pro-angiogenic molecules. Apparently, melanoma cells as compensation produce chemoattractants that stimulate the surrounding stroma in a paracrine manner. These properties of T-cadherin do not allow us to consider it a tumor suppressor because along with the inhibition of vessel growth, T-cadherin stimulates the transcription of genes responsible for survival, migration and invasion of melanoma cells resulting in tumor progression. These data are fundamental for understanding the mechanisms of invasive melanoma progression underlying the dissemination process and low sensitivity to the drug therapy.

## Supplementary Materials

Melanoma cells (B16F10 T−, B16F10 T+, B16F10 T++ clones) were mixed with GFR Matrigels, applied on the Petri dishes and Matrigels were induced to polymerize. After, the preformed drops were covered with complete Matrigels containing the growth factors that were induced to polymerize. RPMI 1640 medium was added into each dish. The drops with invading melanoma cells were filmed during 48 h using a Leica AF6000 microscope. The boundaries between two Matrigels were marked by white lines. The corresponding lifetime observations of invading melanoma cells are provided in the Supplementary Materials.
